# Supplementary material for: Parvimonas micra, an oral pathobiont associated with colorectal cancer, epigenetically reprograms human colonocytes
Source: Gut Microbes. 2023 Oct 16;15(2):2265138. doi: 10.1080/19490976.2023.2265138 (PMC10580862; doi:10.1080/19490976.2023.2265138)
Supplement: Supplemental Material [file KGMI_A_2265138_SM8589.zip › Supplementary material/Supplementary figures and tables legends.docx]

Supplementary Figure 1: Phenotypic and genetic characterization of *P. micra*. (a) Colony appearance of ATCC 33270 (*PmA*) (top) and HHM BlNa17 (*PmB*) (bottom) on blood agar plates: i) after 48 hours of culture under anaerobic conditions at 37°C; ii) when the colonies were removed to observe the hemolytic zone below the colonies; and iii) when colonies were analyzed via the Gram stain. (b) Sedimentation assay performed on *PmA* and *PmB*. On the right, representative pictures of the suspension (top *PmA*, bottom *PmB*) obtained after 2 hours at 4°C without shaking. (c) Clustering of *P. micra* phylotype A and B whole genomes according to the presence (red) or absence (blue) of genes. (d) Adhesion capacity of *P. micra* ATCC 33270-*Pm36* (*PmA*) and HHM BlNa17- *Pm37* (*PmB*) and other oral isolates from phylotype A (*Pm1, Pm10 and Pm24*) or phylotype B (*Pm12*) to Matrigel®. Optical density at 595 nm represents measurement of bacterial adhesion. (e) Adhesion capacity of *PmA*, *Pm1, Pm10, Pm24* from phylotype A and *PmB, Pm12* from phylotype B to human colonic cell lines TC7 after 1 hour of co-culture. Bacterial adhesion was quantified by the analysis of fluorescent images of cells co-cultured with the different isolates and is reported as arbitrary fluorescent units. Mann-Whitney test ****p<0.0001; **p<0.01. (f) Transmission electron microscopy on ultrafine sections of phylotype A isolates *PmA* or *Pm10* (top), and phylotype B isolates *PmB* or *Pm12* (bottom).

Supplementary Figure 2: *Parvimonas* prevalence in feces and tissues of CRC patients. (a) *Parvimonas* prevalence, determined by 16S rDNA sequencing of the V3-V4 region, in controls, adenoma (Ad.), or sporadic CRC patients at early (CRC I&II) and late (CRC III&IV) stages of carcinogenesis in feces, or (b) (c) colonic mucosa. (d) *P. micra* A and B phylotypes prevalence in control and sporadic CRC patients’ feces. (e) *P. micra* A and B phylotypes associated with normal homologous mucosa and tumoral tissue of CRC patients. Prevalence is expressed in percentage of positives samples (number of positives samples/total number of samples x 100). Chi-square test; ****p<0.0001; **p<0.01; *p<0.05).

Supplementary Figure 3: *Parvimonas* carriage in CRC patients’ feces in a large non-EU Cohort. (a) Relative abundance and (b) prevalence of *P. micra* phylotype A and B, in controls and CRC patients in Baxter cohort^62^. Relative abundance is expressed as a percentage of counts (number of sequences assigned to *P. micra* per number of total bacterial sequences X100) and results are presented as mean +/- standard deviation. Prevalence is expressed in percentage of positives samples (number of positives samples/total number of samples x 100). Statistical analysis: (a) Mann-Whitney test, ****p<0.0001; (b) Chi-square test; ****p<0.0001.

Supplementary Figure 4: *P. micra* growth and its effects on cells. (a) *P. micra* oxygen sensitivity. Representative growth curves of *P. micra* ATCC 33270 (*PmA*) under anaerobic (0% O_2_), aerobic (21% O_2_) and hypoxic (2% O_2_) conditions. At different incubation times, the cultures were plated on horse blood plates and returned to anaerobic conditions for 48 hours. CFUs were counted to estimate bacterial viability. The results are expressed as percentage of CFU obtained towards the inoculum grown at 0% O_2_. (b) *P. micra* viability by CFU counts after 48 hours of co-culture with human colonic primary cells in hypoxic conditions (mean +/- SEM). (c), (d), and (e) Quantification of proliferative cells, goblet cells, and cells harboring double-stranded DNA breaks by immunofluorescence using respectively an anti-Ki-67, anti-Muc2, and anti-γH2ax labelling after 48 hours of co-culture with *P. micra* ATCC 33270 (*PmA*), *P. micra* HHM BlNa17 (*PmB*), *F. magna* ATCC 29328 or the *E. coli Pks*+ strain IHE3034 (used as a positive control for double-stranded breaks). NS, non-stimulated. Results are expressed as percentages of positive cells relative to the total number of nuclei and normalized using the non-stimulated sample (NS). Experiments were performed on cells from two donors, with at least 3 experiments per donor. The data are represented as mean +/- SEM. Mann-Whitney test ***p<0.001. (f), (g), and (h) Representative images of Ki-67, Muc2 and γH2ax immunostaining, respectively.

Supplementary Table 1: Description of *P. micra* clinical isolates obtained from several hospitals in Paris and from different infectious sites.

**Supplementary Table 2**: Samples description.

**Supplementary Tables 3**: Excel file with i) the list of differentially expressed genes (DEGs) between 1- *Parvimonas* and 2- *P. micra* phylotype A -negative and -positive patients; ii) Pathway enrichment analysis on RNaseq results; iii) List of differentially methylated regions (DMRs) between *P. micra* -negative and -positive patients.
