# Supplementary material for: Parvimonas micra, an oral pathobiont associated with colorectal cancer, epigenetically reprograms human colonocytes
Source: Gut Microbes. 2023 Oct 16;15(2):2265138. doi: 10.1080/19490976.2023.2265138 (PMC10580862; doi:10.1080/19490976.2023.2265138)
Supplement: Supplemental Material [file KGMI_A_2265138_SM8589.zip › Supplementary material/Supplementary tables and figures.docx]

# a


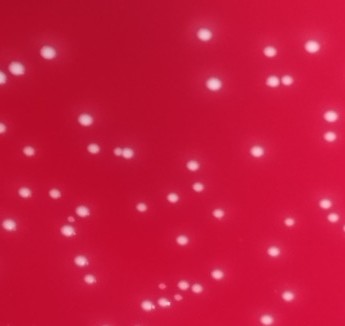

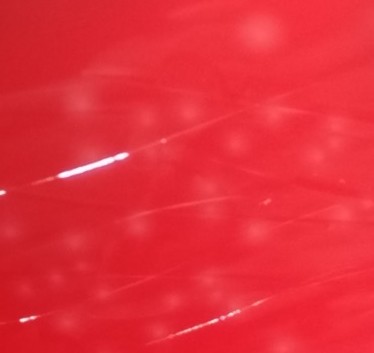

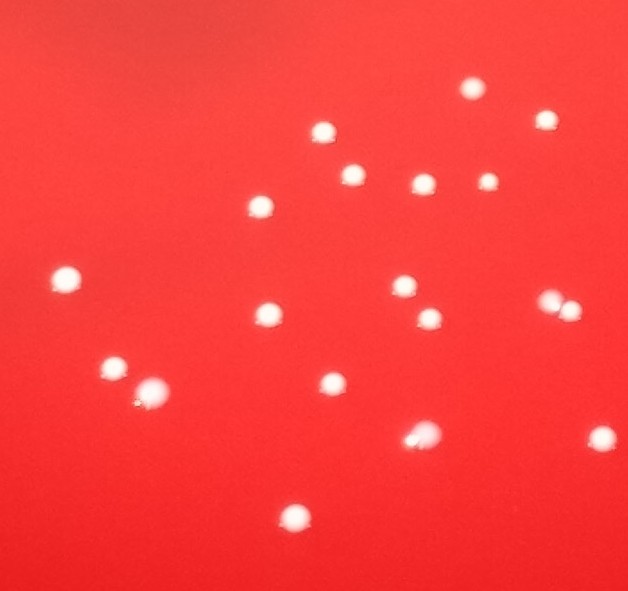

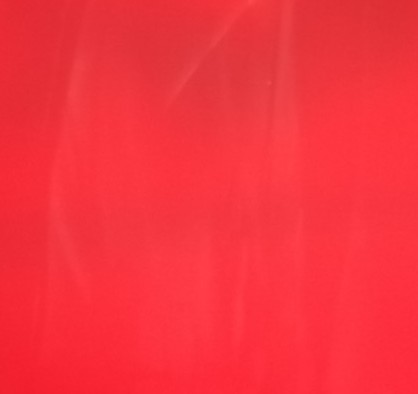


| i. | ii. |
| --- | --- |
|  |  |

*PmA*

*PmB*

**b**

**1.00**

**0.75**

**Sedimentation**

**(OD 600nm)**

**0.50**

**0.25**

*PmA PmB*

*S. flexneri 5a*


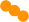

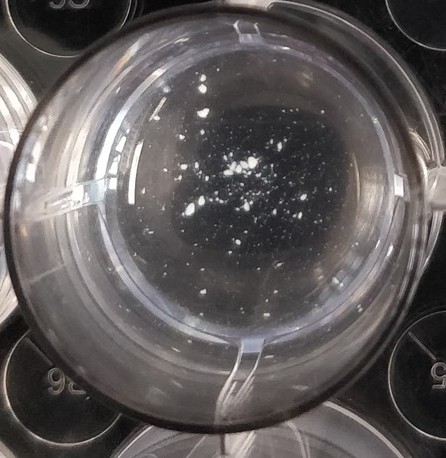

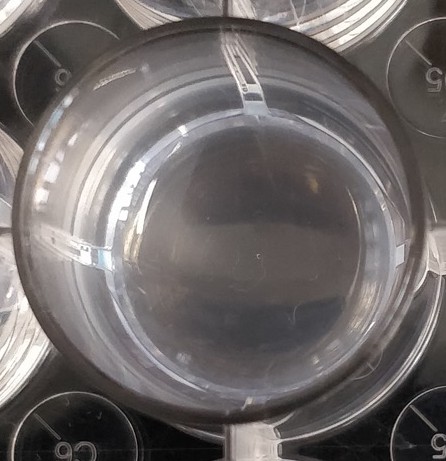


**0 120 240 360**

**Time (min)**

# c


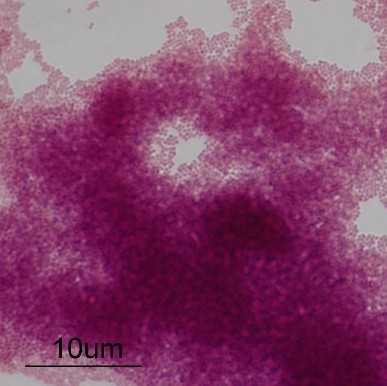

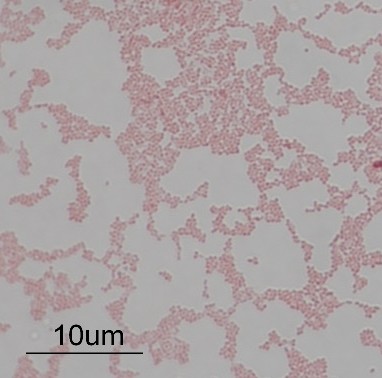


iii.


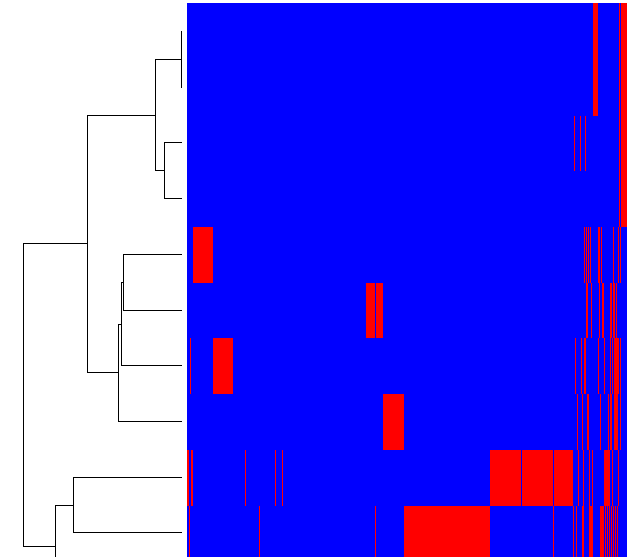

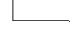

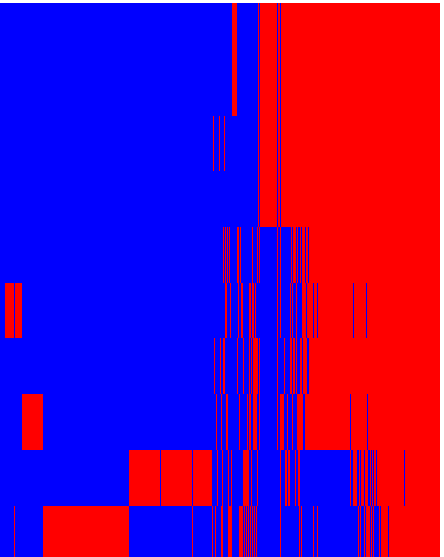


**Phylotype A**

**MGYG-HGUT-01301 ATCC 33270 FDAARGOS-569 NTCL11808**

**13-07-26**

**KCOM1535 / chDC B709 KCOM1037**

**A293 F0139**

**Phylotype B**

# d

**Bacterial adhesion on Matrigel**

*PmA (Pm36)*

#
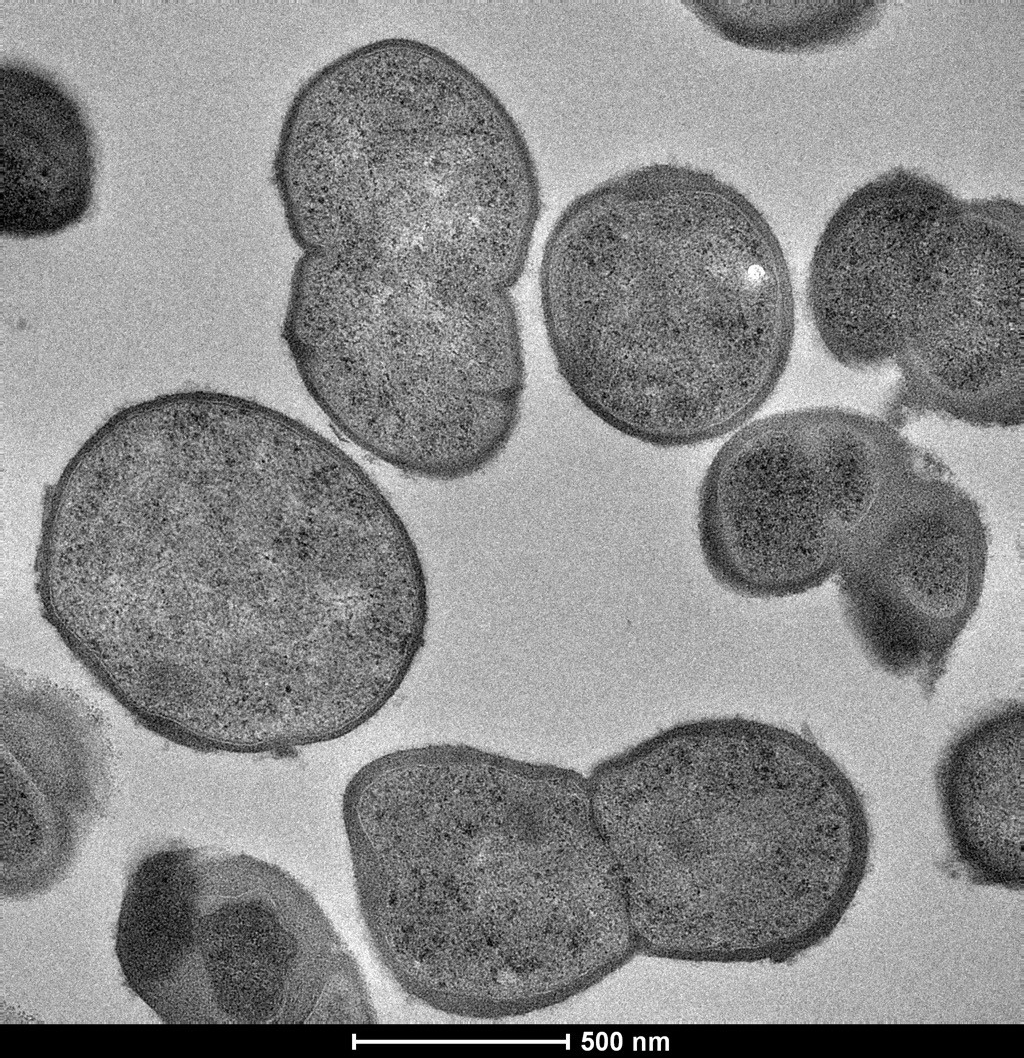
f

### F0440


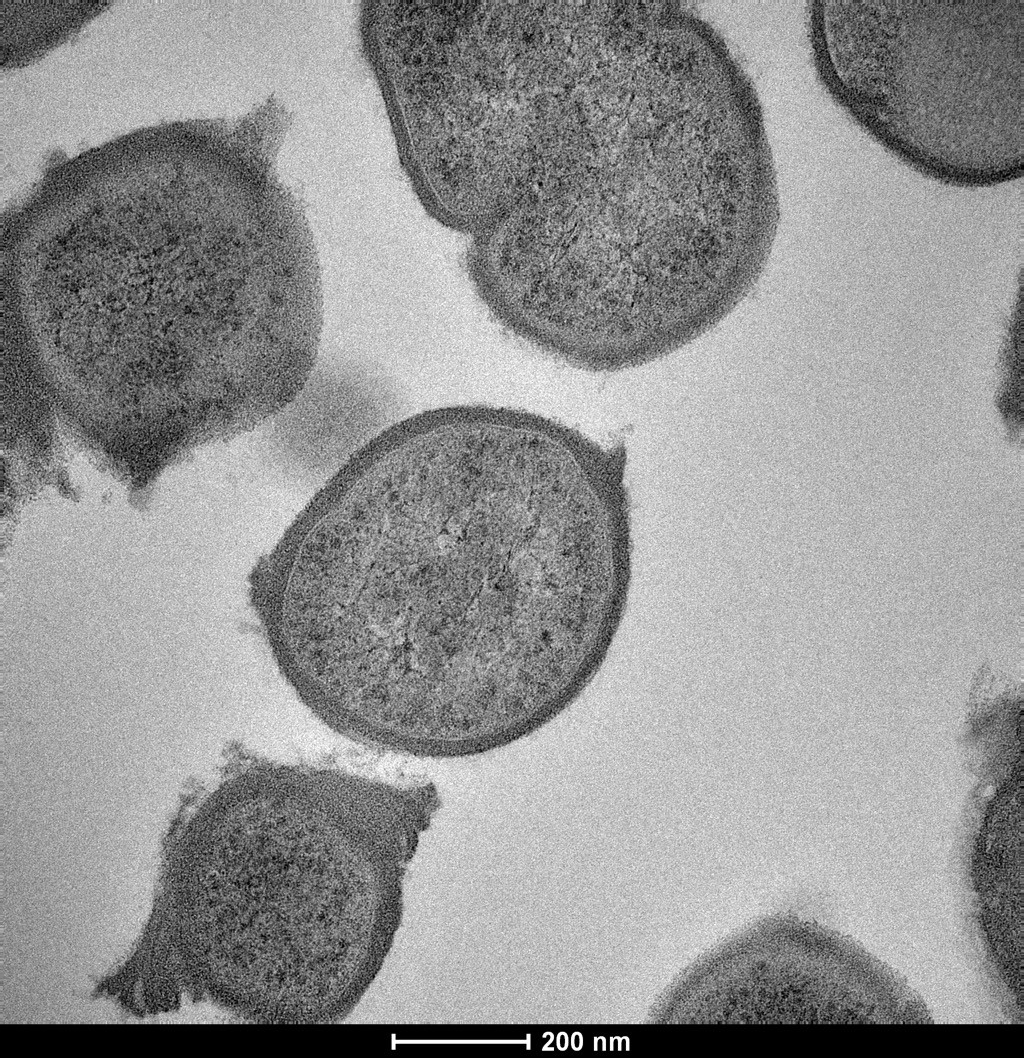
*Pm10*

**2.5** ✱✱

✱✱

**2.0**

**Adhesion (OD 595 nm)**

**1.5**

**1.0**

**0.5**


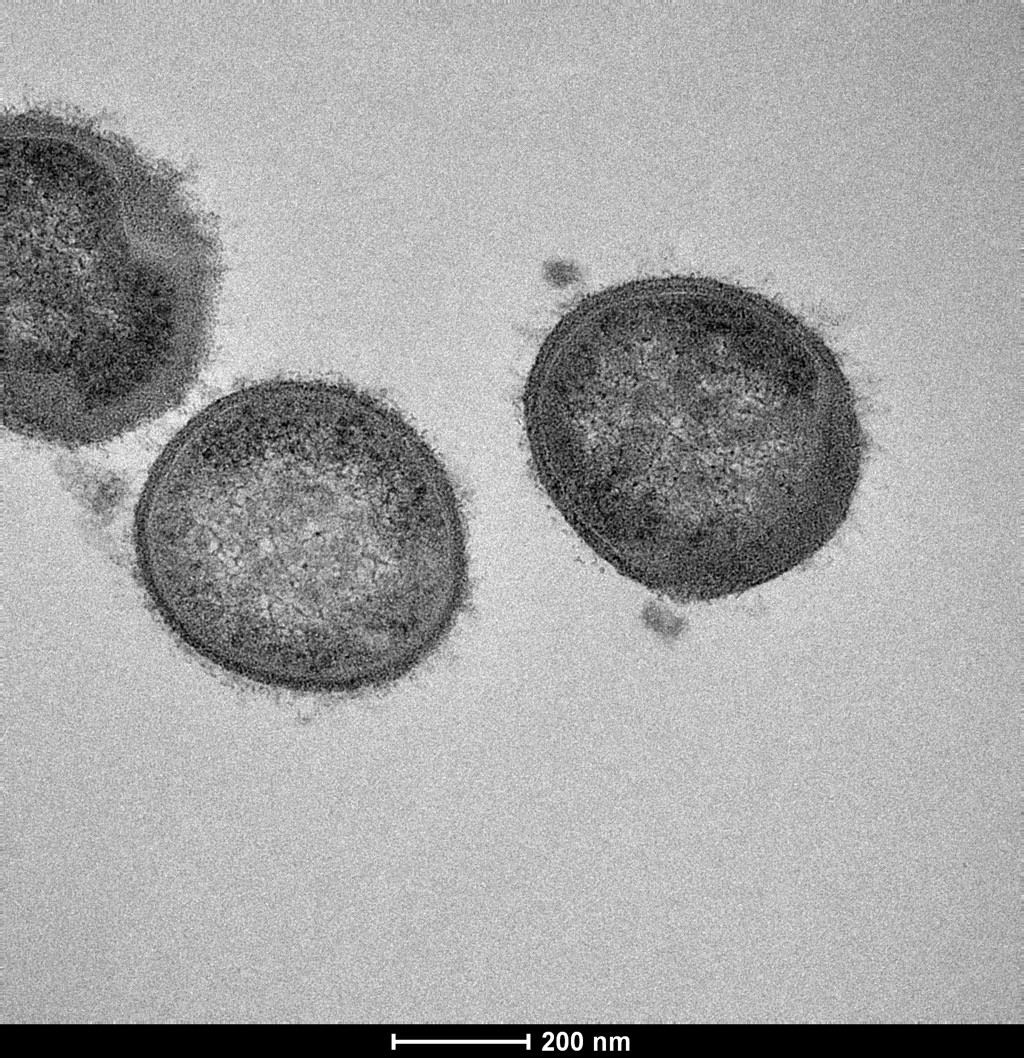

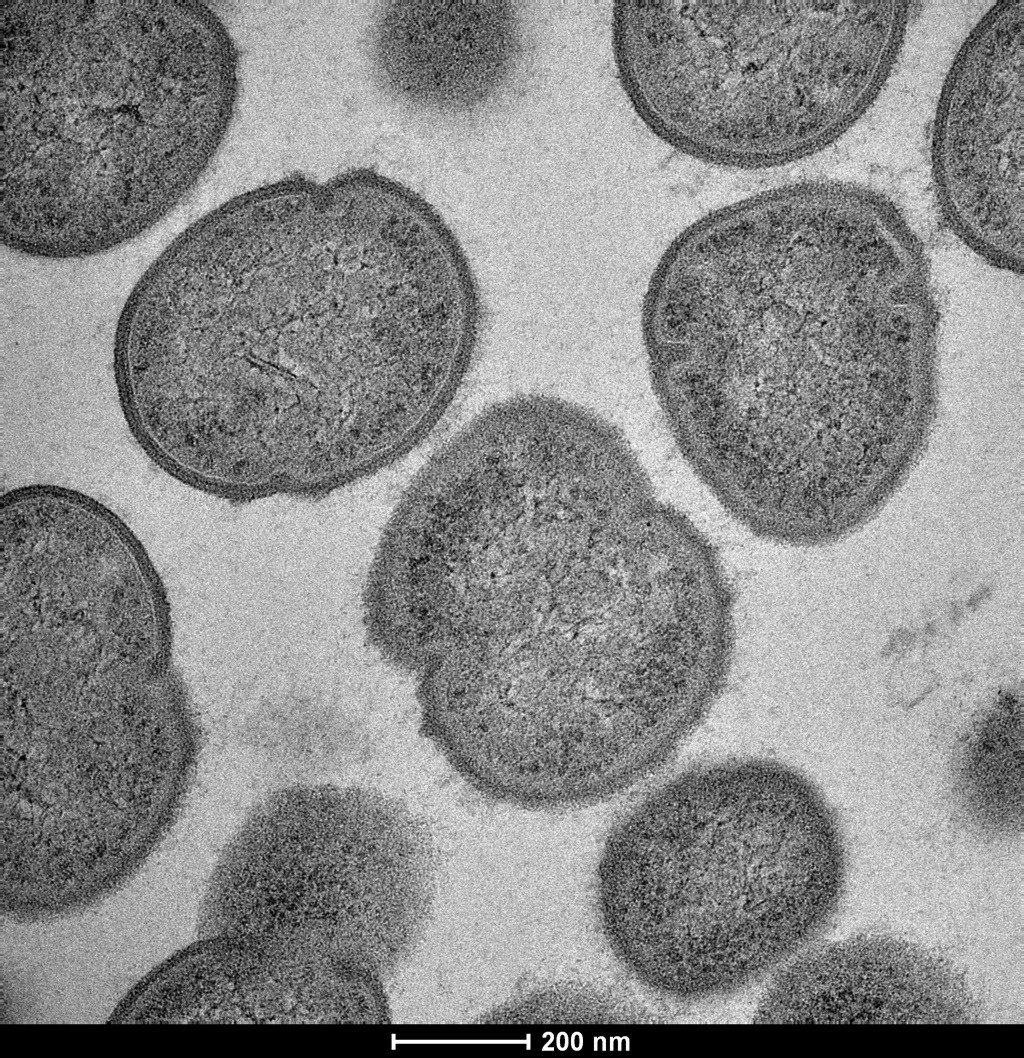
**0.0**

# e

**Bacterial adhesion on TC7 cells**

**10**

✱✱✱✱

✱✱✱✱

**Bacterial adhesion (arbitrary fluorescent units)**

**8**

**6**

**4**

**2**

**0**

*PmB (Pm37)*

*Pm12*

# a

**100**

***Parvimonas***

**positive samples (%)**

**50**

## Feces

✱

**Tumoral tissue**

# b c

**150**

ns

ns

ns

***Parvimonas***

**positive samples (%)**

**100**

**50**

**100**

**80**

***Parvimonas***

**positive samples (%)**

**60**

**40**

**20**

## Tissue

**0 0 0**

ns

✱✱✱

ns

ns

**d Feces**

ns

ns

✱✱

✱

**80**

***Parvimonas***

**positive samples (%)**

**60**

**e**

**80**

Phylotype A

***Parvimonas***

**positive samples (%)**

Phylotype B **60**

## Tissue

ns

ns

✱✱✱✱

✱✱✱✱

Phylotype A Phylotype B

**40 40**

**20 20**

**0 0**

1. **Feces - Phylotype A**

**Feces - Phylotype B**

1. **Feces - Phylotype A**

**Feces - Phylotype B**

**10 10**


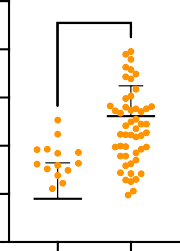


✱✱✱✱

ns

**Relative abundance (%)**

**Relative abundance (%)**

**1 1**

**50 50**

**40 40**

✱✱✱✱

ns

***Parvimonas***

**positive samples (%)**

***Parvimonas***

**positive samples (%)**

**0.1**

**0.01**

**0.001**

**0.1**

**0.01**

**0.001**

**30 30**

**20 20**

**10 10**

**0.0001**

**Controls**

**CRC**

**0.0001**

**Controls**

**CRC**

**0**

**Controls**

**CRC**

**0**

**Controls**

**CRC**

**(n=134) (n=127)**

**(n=134) (n=127)**

**(n=134) (n=127)**

**(n=134) (n=127)**

# a

**1000**

**% of CFU / inoculum**

**100**

***P. micra* O_2_**

### sensitivity

**b**

0% O2

2% O2

21% O2

**2.0×10^7^**

**1.5×10^7^**

**CFU/well**

**1.0×10^7^**

*PmA PmB*

*F. magna*

**10 5.0×10^6^**

**1**

**0 20 40 60 80**

**Time (h)**

**0.0**

**0 48**

### Time of co-culture (h)

1.
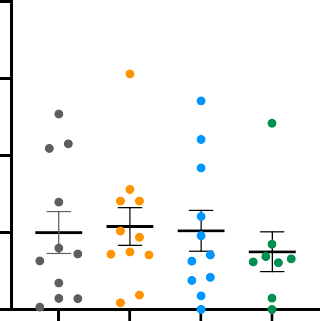
**4 d 4 e 8**


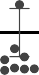


✱✱✱

**Ki-67 positive cells / NS**

**Muc2 positive cells / NS**

γ**H2ax positive cells / NS**

**3 3 6**

**2 2 4**

**1 1 2**

**0 0 0**

# f g h


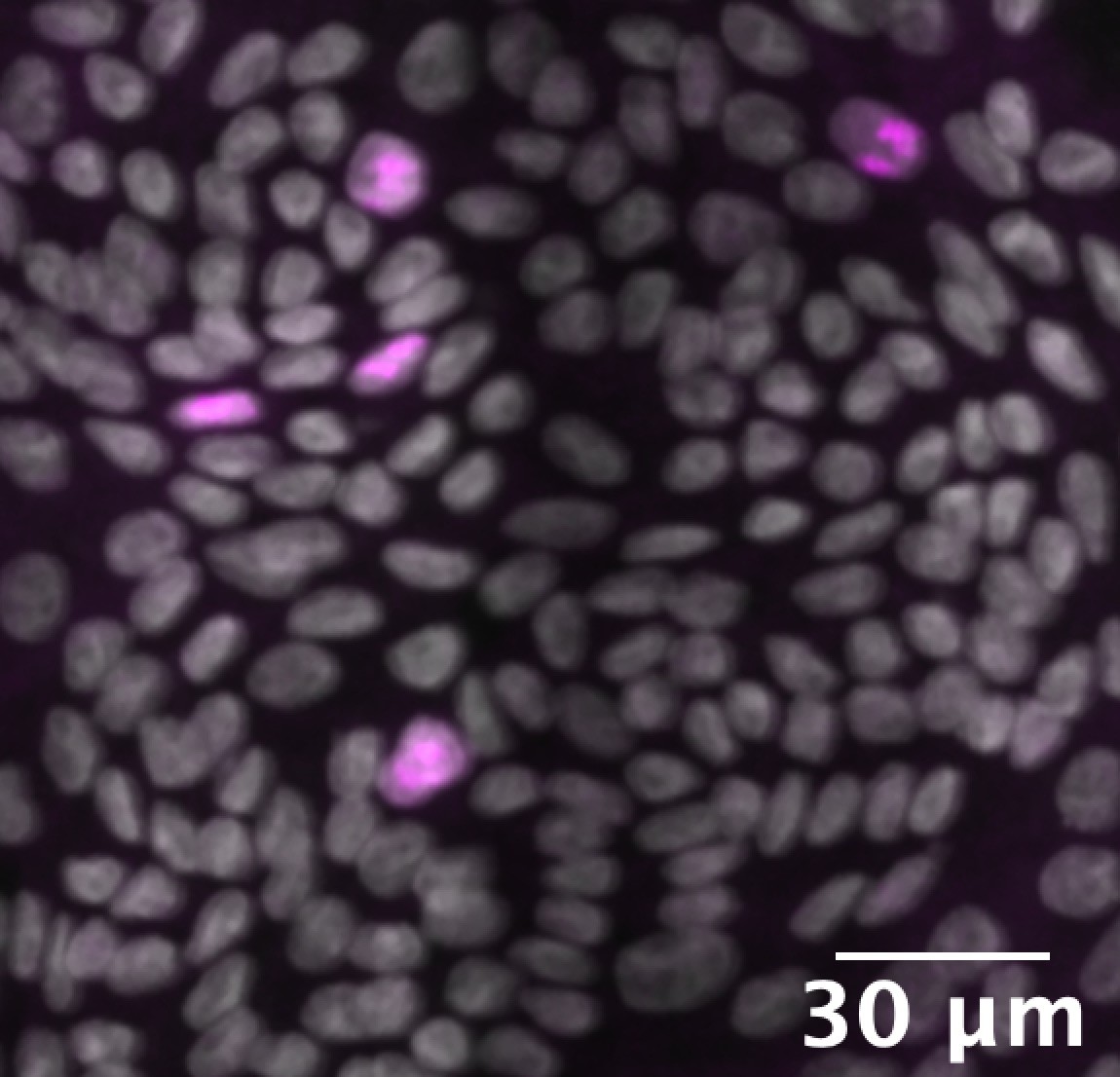

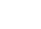

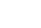

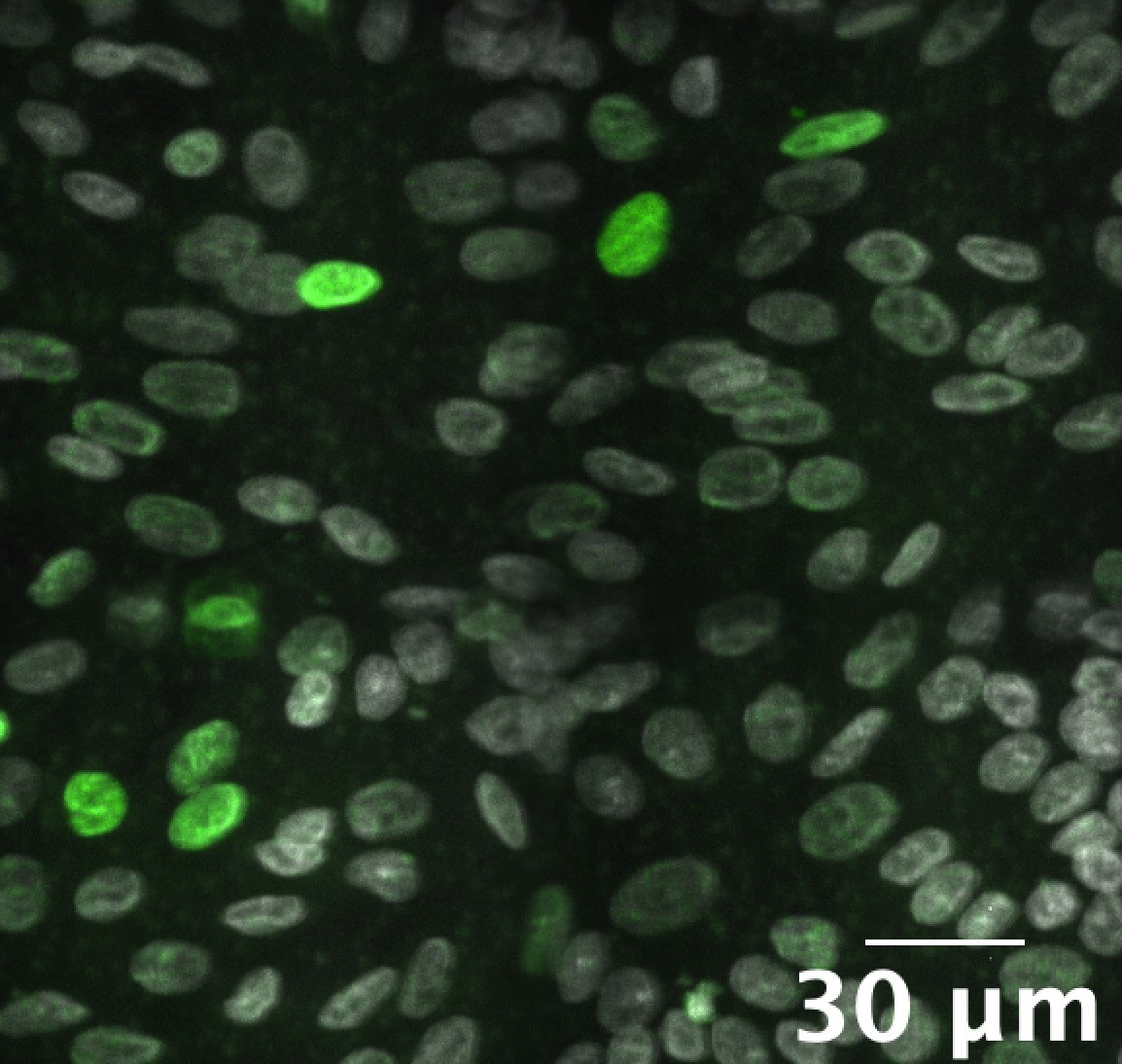

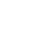

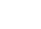

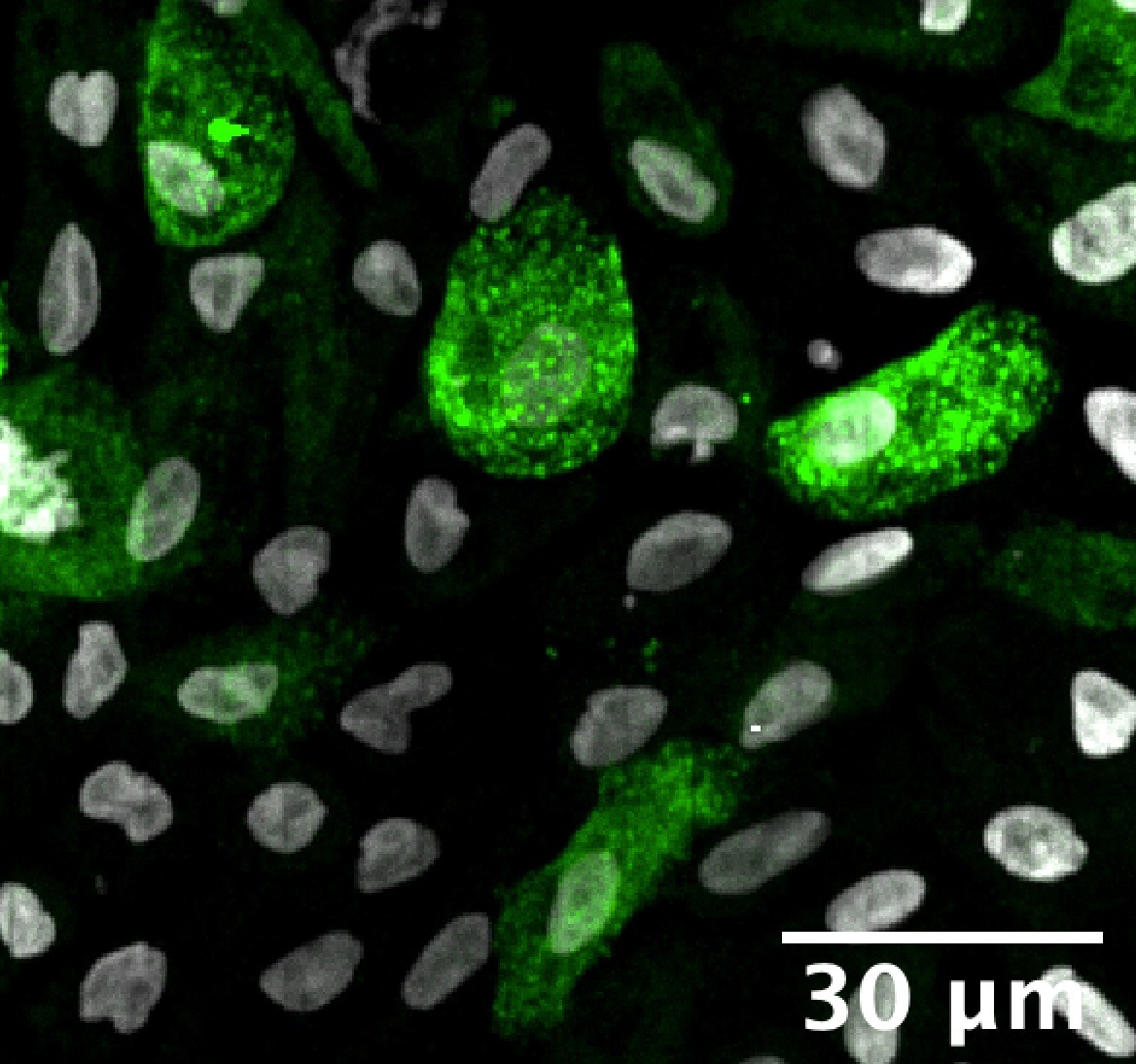

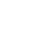

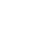


| **Clinical isolate number** | **Origin** | **Source of isolation** | **Hemolysis** | **Colonies Phylogenetic compaction Group** | | **GenBank accession numbers** |
| --- | --- | --- | --- | --- | --- | --- |
| PM 1 | Pitié Salpêtrière | Dental cellulite | + | - A1 | | OM287472 |
| PM 10 | Pitié Salpêtrière | Dental cellulite | + | - A1 | | OM287474 |
| PM 14 | Pitié Salpêtrière | Cerebral abscess | + | - A1 | | OM287469 |
| PM 15 | Pitié Salpêtrière | Cerebral abscess | + | - A2 | | OM287478 |
| PM 17 | Pitié Salpêtrière | Cerebral abscess | + | - A1 | | OM287473 |
| PM 19 | Pitié Salpêtrière | Cervical abscess | + | - A1 | | OM287466 |
| PM 20 | Pitié Salpêtrière | Cervical adenopathy | + | - | A1 | OM287465 |
| PM 24 | Pitié Salpêtrière | Lower canaliculus | + | - | A1 | OM287467 |
| PM 26 | Pitié Salpêtrière | Cerebral abscess | + | + | A1 | OM287468 |
| PM 28 | Cochin | Femur | + | + | A1 | OM287464 |
| PM 3 | Pitié Salpêtrière | Cerebral abscess | + | - | A1 | OM287476 |
| PM 31 | Henri Mondor | Empyema brain | + | - | A1 | OM287463 |
|  |  | abscesses |  |  | |  |
| PM 32 | Cochin | Hip articular | + | - A1 | | OM287471 |
| PM 34 | Cochin | Hip | + | - A1 | | OM287470 |
| PM 35 | Henri Mondor | Hemoculture | + | - A1 | | OM287462 |
| **PM 36 / ATCC 33270 / *PmA*** | CIP Pasteur | Purulent pleurisy | + | - | A1 | OM287461 |
| PM 38 | CIP Pasteur | Abdominal wound | + | - | A1 | OM287460 |
| PM 4 | Pitié Salpêtrière | Cerebral abscess | + | - | A2 | OM287477 |
| PM 40 | Henri Mondor | Hemoculture | + | - | A1 | OM287459 |
| PM 6 | Pitié Salpêtrière | Cerebral abscess | + | - | A1 | OM28747 |
| PM 25 | Pitiée Salpêtrière | Cerebral abscess | - | + | B2a | OM287480 |
| PM 33 | Cochin | Knee joint | - | - | B2a | OM287479 |
| PM 12 | Pitié Salpêtrière | Maxillary sinus | - | + | B2b | OM287481 |
| PM 2 | Pitié Salpêtrière | Cervical collection | - | - | B2b | OM287484 |
| PM 29 | Cochin | Urinary tract | - | + | B2b | OM287482 |
| PM 30 | Cochin | Femur | - | + | B2b | OM287483 |
| **PM 37 / HHM** |  |  |  |  |  |  |
| **BlNA17 /** | Henri Mondor | Hemoculture | - | + | B2b | OM287485 |
| ***PmB*** |  |  |  |  |  |  |
| **PmG5** | Henri Mondor | Colonic tumoral  biopsy | + | - | A | OQ45037 |

**Supplementary Table 1**: Description of *P. micra* clinical isolates obtained from several hospitals in Paris and from different infectious sites.

### Samples Feces

**n= 166**

**Tissue (tumor and homologous) n=71**

*Status* Control n= 88; CRC n= 78 CRC n=71

*Tumor localization* Right or transversal colon n= 27 Sigmoid or left Colon n= 48, ind=3

Right or transversal colon n= 35 Sigmoid or left Colon n= 41

*TNM stages* I or II n= 27;

III or IV n= 48, ind=3

I or II n= 40; III or IV n=31

*Gender (Female/male)* F: n= 70; M: n= 76

F: n= 23; M: n=48

*Ages (mean +/- SEM)* Control: 60.48 +/- 1.13 CRC: 66.23 +/-1.60

CRC: 65.46 +/- 1.22

*BMI (kg/m2) mean +/- SEM* Control: 25.18 +/- 0.4

CCR: 25.67 +/- 0.81

CRC: 25.2 +/- 0.5

*Cumulative methylation index (Negative/Positive)*

N: n= 119; P: n= 43 N: n= 8; P: n= 7

**Supplementary Table 2:** Samples description. Ind, indetermined.
